# Oral Health Status, Health Behaviour and Treatment Needs of Patients Undergoing Cardiovascular Surgery

**DOI:** 10.21470/1678-9741-2017-0137

**Published:** 2018

**Authors:** Ansul Kumar, Arpita Rai

**Affiliations:** 1 Rajendra Institute of Medical Sciences (RIMS) Ranchi, Jharkhand, India.; 2 Faculty of Dentistry, Jamia Millia Islamia, New Delhi, India.

**Keywords:** Oral Health, Cardiovascular Surgical Procedures, Oral Hygiene

## Abstract

**Objective:**

The aim of the present study was to assess the oral health status and treatment needs of cardiovascular surgery patients. Second, the awareness of cardiovascular surgery patients regarding the association between oral health and heart disease was considered.

**Methods:**

Assessment of oral health status, oral hygiene practices and treatment needs of 106 hospitalized patients in preparation for cardiovascular surgery. Patients were interviewed using a structured questionnaire designed for this study and oral examination was carried out by a dentist.

**Results:**

The oral hygiene practices of the study cohort were not up to the standard. Patients' awareness of infective endocarditis was poor. Approximately 68% patients experienced dental caries as decayed teeth or missing teeth due to caries and filled teeth. The mean plaque index in the study group was 1.25. In this study cohort, the mean probing depth of periodontal pockets was 5.7±1.3, whereas the mean number of teeth with periodontal pockets > 6 mm was 0.5±0.9. A total of 84 (74.2%) of the patients required dental treatment.

**Conclusion:**

The principal finding in this study was that patients with heart disease had poor oral health. This study also highlights the importance of better interaction among all healthcare professionals to integrate oral health as part of comprehensive inpatient healthcare.

**Table:** 

Abbreviations, acronyms & symbols	
CHD	= Coronary heart disease
CVDs	= Cardiovascular diseases
CVS	= Cardiovascular surgery
DMFT	= Decayed, missing and filled teeth
IE	= Infective endocarditis
PI	= Plaque index
WHO	= World Health Organization

## INTRODUCTION

Cardiovascular diseases (CVDs) are the most important cause of mortality in India. The age-standardized Global Burden of Disease study estimates that 24.8% of all deaths in India is attributed to CVD^[1]^. Coronary heart disease (CHD) and stroke are responsible for > 80% of CVD deaths^[2]^.

Cardiovascular surgery (CVS) encompasses several surgical procedures like myocardial revascularization, valve repair or replacement, aortic diseases, correction of congenital heart disease, cardiac pacemaker implantation and heart transplant.

In 1909, Horder stressed that "oral sepsis" was the prime reason for the genesis of infective endocarditis (IE)^[3]^. The introduction of bacteria from oral foci of infection into the bloodstream can lead to a transient bacteremia, enabling the adhesion of microorganisms to previously compromised cardiac tissues. CHD is considered an inflammatory disorder^[4]^. Dental infections, particularly periodontal, have been associated with atherosclerosis^[5-8]^. Infection disturbs the coagulation mechanisms and activates pathologic processes in the coronary arteries. There may be damage to the endothelium and initiation of a fibro-proliferative process in the artery, leading to atherosclerosis^[9]^.

Several studies have shown a positive association between dental health and heart disease, but little is known about the oral health status in CVS patients. In addition, the behavioural aspects and attitudes regarding the oral health of these patients and their treatment needs remains a scarcely explored subject. The aim of the present study was to assess the oral health status and treatment needs of CVS patients. Second, the awareness of CVS patients regarding the association between oral health and heart disease was considered.

## METHODS

This is a cross-sectional, observational study in which patients admitted for elective cardiac surgery at a public hospital were included. The study protocol was approved by the institutional ethics committee, PGIMER Dr. RML Hospital, New Delhi. Informed written consent was taken by patients.

A total of 123 patients diagnosed with cardiovascular disease and hospitalized in preparation for CVS were approached to participate in the study, of which 106 patients volunteered to participate. Exclusion criteria included patients who needed emergency cardiac surgery or those whose heart disease was so severe that the clinical oral examination could not be safely done. The study sample ranged from 12-73 years age and male:female was 3:1.2. The CVD distribution of the study population is presented in Table 1.

**Table 1:** Distribution of the studied population according to the cardiovascular procedure undergone by them.

S. No	Cardiovascular procedure	Males	Females	Total
1	Bypass surgery	23	7	30 (28.3%)
2	Valve surgery	44	19	63 (59.4%)
3	Congenital corrective surgery	9	4	13 (12.2%)
	Total	76	30	106

The study participant's demographical data, CVD diagnosed and any other systemic illnesses were recorded. They were interviewed using a structured questionnaire designed for this study, which included aspects as patient knowledge about IE, oral symptoms experienced by the patient, satisfaction with oral health quality and dental aesthetics, and if they carried an oral hygiene kit with them in the hospital. Questions also included the history of past dental treatment, their oral hygiene practices and other behavioural habits. Patients were referred to the dental department of PGIMER Dr. RML Hospital, New Delhi, where the dentist evaluated them. The recommendations of the World Health Organization (WHO) were followed in the clinical assessment. The oral examination included scores for caries (Decayed, Missing and Filled Teeth - DMFT index), oral hygiene (plaque index) and periodontal status using a standard probe. Data were tabulated, and statistical analysis was done.

## RESULTS

In this study, composed of 76 males and 30 females posted for cardiac surgery, the self-perceived quality of oral health was excellent in 10 (9.4%), good in 41 (38.6%), fair in 38 (35.8%) and bad in 17 (16%). Approximately 45% (48 patients) reported the presence of at least one oral symptom such as dental pain, decay, tooth sensitivity, etc. Although 35 (33%) patients were aware of an association between oral health and heart disease, none of them were aware of IE. A total of 87 patients reported that they took their oral hygiene kits to the hospital. Approximately 60% patients reported having had a previous dental visit whereas only 18.6% of them had undergone oral prophylaxis.

### Oral Health Behaviour and Practices

Approximately two-thirds of the patients did not brush their teeth daily, and only 10.4% brushed twice a day. Twenty-three percent of patients reported using cleaning aids other than toothpaste, such as tooth powder, charcoal, burnt tobacco, etc. Cleaning the interdental spaces using a floss or toothpick was uncommon, although the use of toothpick was reported more often than floss. Table 2 shows the main results of oral hygiene practices and behaviour of CVS patients.

**Table 2:** Oral hygiene practices and behaviour of the studied population.

S. No	Oral hygiene practices	Number of patients (%)
1	Tooth brushing	
	Once a day	34 (32.1%)
	Twice a day	11 (10.4%)
	None	61 (57.5%)
2	Use of mouthwash	
	Yes	15 (14.2%)
	No	91 (85.8%)
3	Use of dental floss	
	Yes	7 (6.6%)
	No	99 (93.4%)
4	Use of toothpick	
	Yes	27 (25.4%)
	No	79 (74.6%)
5	Cleaning aid for brushing	
	Toothpaste	71 (66.9%)
	Tooth powder	16 (15%)
	Charcoal	8 (7.5%)
	Crystal salt	4 (3.7%)
	Tobacco	5 (4.7%)
	Any other	2 (1.8%)

### Caries Experience - DMFT

Approximately 68% of the patients experienced dental caries as decayed teeth or missing teeth due to caries and filled teeth. Approximately 41 (38.6%) of the study subjects had one or more decayed teeth, 56 (53%) had missing teeth due to caries and 18 (16.9%) had a filling. The mean DMFT score was 1.28 (SD=9.15) with predominance from missing and decayed components. Table 3 shows the details of DMFT score among the study population.

**Table 3:** Details of DMFT score among the studied population.

Variables	
DMFT (mean ± SD)	1.28±9.15
DT (median, range)	3 (0-14)
MT (median, range)	4 (0-32)
FT (median, range)	1 (0-9)

DMFT=number of decayed, missing and filled teeth (caries index); DT=decayed teeth; FT=filled teeth; MT=missing teeth

### Plaque Index

The plaque index (PI) assesses the accumulation of plaque to the naked eye, without using plaque disclosing agents. The score ranges from 0 (no plaque) to 3 (a lot of plaque in teeth and gingival margin). Each tooth was examined at four points and the highest score was determined^[10]^. The mean PI in the study group was 1.25. Table 4 shows the distribution of PI scores in the study population.

**Table 4:** Distribution of plaque index scores in the studied population.

S. No	Rating	Score	Number of patients
1	Excellent	0	__
2	Good	0.1-0.9	17 (16.03%)
3	Fair	1.0-1.9	65 (61.32%)
4	Poor	2.0-3.0	24 (22.64%)

### Periodontal Status

A standard mm-graded probe was used to assess the pocket depth. The pocket > 6 mm was defined as deep periodontal pocket. In this study cohort, the mean probing depth of periodontal pockets was 5.7±1.3, whereas the mean number of teeth with periodontal pockets > 6 mm was 0.5±0.9.

### Dental Treatment Needs

Study participants were assessed for the need of dental treatment. Approximately only 21% of the patients did not require dental treatment. Table 5 describes the specific treatment needs of the population studied.

**Table 5:** Dental treatment needs of the studied population.

S. No	Dental treatment needs	Number of patients (%)
1	No treatment required	23 (21.7%)
2	Periodontal treatment	73 (68.8%)
3	Restorative and endodontic treatment	41 (38.6%)
4	Exodontia and other surgical management	18 (16.9%)
5	Prosthodontic rehabilitation	56 (53%)

## DISCUSSION

The present observational study evaluated the oral health status of preoperative patients admitted to the department of cardiothoracic vascular surgery. The WHO's Global Oral Health Program emphasized the significance of escalating efforts to increase awareness of oral health worldwide as a major component of general health and quality of life^[11]^. Among hospitalized patients, it is generally observed that oral healthcare is often ignored due to the burden of other health -related duties and the priority of medical care.

The self-perception of the oral health quality of the majority of patients did not coincide with the dental treatment needs of the studied population, since only 23 (21.7%) patients did not show need of dental treatment. Similar results have been reported by Amaral et al.^[12]^. This emphasises the lack of awareness of dental problems among study participants. It could be possible that patients would not pay attention to their oral problems due to the life-threatening nature of their cardiac condition.

Knowledge of the study participants regarding the existence of association between oral health and heart disease was fair, but none of the participants were aware of the risk of complications. Similar findings have been reported by several authors in children^[13,14]^. This presents a challenge especially among those who have poor oral health. There is an urgent need to educate patients with heart disease regarding the two-way relationship between oral health and systemic health.

In our study, it was surprising to note that the number of patients who never brushed was significantly high. The percentage of patients who brushed regularly once a day (32.1%) was lower than the findings of other studies^[13,15]^. Teeth must be examined and cleaned professionally by a dentist in patients with cardiac disease. The oral hygiene practices of these patients, such as the use of charcoal, tobacco and salt as a cleaning aid, could explain the high prevalence of edentulism in this study cohort.

The present study shows that a significant part of the studied population had experienced caries as evident by the DMFT score. A direct comparison of the DMFT score of the patients investigated in this study with other studies is limited, since different age groups were included. A study by Rai et al.^[16]^ reported experience of dental caries in 70.3% of hospitalized patients. Higher DMFT scores have been reported elsewhere in cardiac patients^[13,17]^. The higher number of patients showing fair to poor rating in PI signifies a poor oral hygiene prevalence among these patients. Meurman et al.^[18]^ have reported that, on average, 0.4 surfaces of a tooth are covered by plaque. Lower median PI (0.75) has been reported in acute coronary syndrome^[17]^.

### Limitations

This study has some limitations that must be considered while interpreting the results: is a single-centre study and therefore the sample size may not be representative of all the hospitalized patients. We recommend a multicentre study to eliminate this bias.

## CONCLUSION

The principal finding in this study was that patients with heart disease had poor oral health. Therefore, the value of targeted oral health education protocols must be emphasized. There is a need for intensive efforts in the dental profession to highlight the need for regular dental care for patients at risk for heart disease. The key to oral diseases prevention is consistent daily home care to maintain good oral hygiene in combination with regular dental care by the dentist. This study also highlights the importance of better interaction among all healthcare professionals to integrate oral health as part of comprehensive inpatient healthcare.

**Table:** 

Authors' roles & responsibilities	
AK	Substantial contributions to the conception or design of the work; or the acquisition, analysis, or interpretation of data for the work; agreement to be accountable for all aspects of the work in ensuring that questions related to the accuracy or integrity of any part of the work are appropriately investigated and resolved; final approval of the version to be published
AR	Substantial contributions to the conception or design of the work; or the acquisition, analysis, or interpretation of data for the work; drafting the work or revising it critically for important intellectual content; final approval of the version to be published
